# Compensatory attenuation of cortical apoptosis by SK2 downregulation following ketamine anesthesia

**DOI:** 10.3389/fphar.2026.1761187

**Published:** 2026-03-12

**Authors:** San Huang, Li Li, Yajuan Wang, Ming Xu, Yingwei Wang, Qi Wang

**Affiliations:** 1 Department of Anesthesiology, Huashan Hospital, Fudan University, Shanghai, China; 2 Department of Anesthesiology, Affiliated Hospital of North Sichuan Medical College, Nanchong, Sichuan, China

**Keywords:** compensatory mechanisms, ketamine, neuronal apoptosis, SK channel, ubiquitin-proteasome system

## Abstract

**Introduction:**

A single exposure to general anesthetic can induce acute increase in neuronal apoptosis of the neonatal brain; however, how the brain counteracts the anesthetic-induced neurotoxicity remains unknown. The aim of this study is to explore how the neonatal cerebral cortex responds to anesthetic-induced neuronal apoptosis and the underlying mechanisms involved.

**Materials and Methods:**

Postnatal day 7 rats received intraperitoneal ketamine injections. Apoptotic neurons in the primary somatosensory cortex (S1) were quantified via immunohistochemistry. Whole-cell patch-clamp recordings were performed to assess neuronal activity of pyramidal neurons, including small conductance Ca^2+^-activated potassium (SK) channel-mediated medium afterhyperpolarization (mAHP) currents and spike frequency. SK2 expression was analyzed via Western blot, with genetic manipulation (overexpression/knockdown) to investigate its role in apoptosis regulation. SK2 ubiquitination and the ubiquitin-proteasome system (UPS) involvement were probed by co-immunoprecipitation and proteasomal inhibitor MG132.

**Results:**

Ketamine induced an acute surge in S1 neuronal apoptosis (mean ± SEM, control vs. ketamine, 1386.11 ± 253.63/mm^3^ vs. 2229.07 ± 239.78/mm^3^, *P* = 0.0247), followed by a significant reduction at 24 h post-anesthesia (1281.35 ± 316.07/mm^3^ vs. 554.24 ± 59.43/mm^3^, *P* = 0.0417). This compensatory anti-apoptotic response coincided with attenuated SK channel-mediated mAHP currents (487.33 ± 38.00 pA vs. 355.33 ± 23.49 pA, *P* = 0.0058), which consequently enhanced neuronal spike frequency. Concurrently, both total (0.75 ± 0.04, *P* = 0.0156) and surface (0.76 ± 0.02, *P* = 0.0078) expression of SK2 channels were decreased in S1. SK2 overexpression reversed elevated neuronal excitability and blocked apoptotic reduction, while SK2 knockdown mimicked the pro-excitability and anti-apoptotic effects. SK2 downregulation relied on UPS-dependent degradation: MG132 restored SK2 levels, normalized spike frequency, and inhibited apoptotic reduction.

**Conclusion:**

The developing cortex compensates for ketamine-induced neuronal apoptosis by suppressing subsequent physiological apoptosis. This anti-apoptotic response is critically mediated by increased neuronal activity, driven by UPS-dependent SK2 downregulation.

## 1 Introduction

The potential neurodevelopmental risks of general anesthetics in early life represent a significant concern in pediatric anesthesiology, drawing attention from clinicians and families alike (Ing and Bellinger, 2022; Jevtovic-Todorovic, 2013; Jevtovic-Todorovic et al., 2013; Nasr and Davis, 2015; Servick, 2014). While prospective epidemiological studies suggest that multiple exposures may correlate with subtle deficits in processing speed or motor skills, single anesthesia in otherwise healthy children appears to have minimal impact on intelligence measures (Ing et al., 2021; McCann et al., 2019; Sun et al., 2016; Warner et al., 2018). Despite ongoing clinical debate, extensive preclinical evidence consistently demonstrates that general anesthetics can disrupt brain development, with the severity of effects dependent on dosage, duration, and exposure frequency (Alvarado et al., 2017; Li et al., 2025; Qiu et al., 2024; Wang et al., 2022; Wang et al., 2017).

A critical finding is that nearly all clinically used anesthetics trigger neuronal apoptosis in the developing mammalian brain (Areias et al., 2023; Creeley and Olney, 2010; Jevtovic-Todorovic et al., 2013; Li et al., 2025). Vulnerability peaks during the brain growth spurt—corresponding to the first two postnatal weeks in rodents—when physiological apoptosis actively refines neuronal circuits by eliminating surplus neurons (Ikonomidou et al., 1999; Southwell et al., 2012; Wang et al., 2017; Wong and Marín, 2019). This developmental pruning ensures optimal network size and function (Dekkers et al., 2013). Although even a single anesthetic exposure can significantly increase neuronal apoptosis in the developing brain, the resulting reduction in neuronal density has been shown to be transient (Chen et al., 2016; Jiang et al., 2016), suggesting the existence of compensatory mechanisms that restore neuronal numbers over time. However, the underlying processes facilitating this recovery remain poorly understood.

Neuronal activity is a pivotal regulator of apoptosis and survival in developing cortical circuits (Blanquie et al., 2017a; Blanquie et al., 2017b; Priya et al., 2018). Elevated activity promotes survival, whereas suppressed activity exacerbates cell death (Schroer et al., 2023; Wong Fong Sang et al., 2021). During early development, the activity of cortical pyramidal neurons can also regulate interneuron apoptosis (Wong et al., 2018). Enhancing pyramidal neuron activity suppresses physiological interneuron apoptosis and increase neuronal density (Wong et al., 2018). Critically, we previously demonstrated that chemogenetic manipulation of neuronal activity bidirectionally regulates neonatal cortical neurons’ susceptibility to anesthetic-induced apoptosis: increasing neuronal activity reduced ketamine-induced cortical apoptosis, whereas inhibiting activity exacerbated it (Wang et al., 2017). These findings led us to hypothesize that the neonatal cortex compensates for anesthesia-induced apoptosis by upregulating intrinsic neuronal activity.

To test this, we quantified neuronal apoptosis in the neonatal rat primary somatosensory cortex (S1) at multiple timepoints following a single ketamine exposure, simultaneously assessing neuronal activity. Apoptosis peaked at 6 h post-exposure but fell below baseline levels by 24 h. This compensatory decrease in apoptosis coincided with elevated pyramidal neuron activity, evidenced by increased spike frequency and reduced medium afterhyperpolarization (mAHP) currents. Given the key role of small-conductance calcium-activated potassium (SK1-3; also known as KCNN1-3 or KCa2.1-2.3) channels in regulating spike frequency via mAHP currents in pyramidal neurons (Gill and Hansel, 2020; Villalobos et al., 2004), we investigated their involvement. SK2 expression in S1 was significantly downregulated 24 h post-ketamine. SK2 overexpression increased mAHP currents, dampened neuronal activity, and impeded the compensatory reduction in apoptosis. Conversely, SK2 knockdown increased spike frequency and decreased apoptosis in controls. Mechanistically, enhanced SK2 ubiquitination promoted its endocytosis and proteasomal degradation (Müller et al., 2018; Sun et al., 2020; Sun et al., 2015). Pharmacological proteasome inhibition (MG132) rescued SK2 expression, normalized mAHP currents and spike frequency, and blocked the compensatory apoptosis reduction.

Collectively, these results identify a novel neuroprotective pathway: following ketamine-induced apoptosis, neonatal S1 upregulates neuronal activity via SK2 ubiquitination and degradation, leading to reduced mAHP, elevated excitability, and ultimately, limited apoptotic death. This study reveals SK2 channels as critical mediators of activity-dependent compensation after developmental anesthetic exposure, illuminating the brain’s intrinsic capacity to counteract neurotoxic insults.

## 2 Materials and methods

### 2.1 Animal anesthesia and treatment

Sprague-Dawley rats on postnatal day 0–10 (P0 - P10) were used. All animal procedures complied with the National Institutes of Health Guide for the Care and Use of Laboratory Animals and were approved by the Ethical Committee for Animal Research of Fudan University. All rats were reared under a 12 h light/12 h dark cycle in temperature- and humidity-controlled rooms. Both male and female pups were used.

Littermate pups were randomly assigned to control or ketamine anesthesia groups. Rats in the anesthesia group were injected intraperitoneally (i.p.) with ketamine at doses of 60 mg/kg, and control littermates received an equal volumes of phosphate buffer solution (PBS). The loss of righting reflex (LORR) was quickly induced by ketamine administration and it would maintain for almost 2–3 h without significantly affecting normal oxygenation and respiration as described previously (Wang et al., 2017). After PBS or ketamine administration, pups were housed in temperature-controlled chambers (each group separately) to maintain their normal body temperature. For the MG132 studies, P7 pups were intraperitoneally treated with 0.5 mg/kg MG132 (Sigma-Aldrich) or the vehicle (15 μL DMSO in 1 mL PBS) 30 min prior to ketamine anesthesia.

### 2.2 Electrophysiological recordings in acute brain slices

Rats were deeply anesthetized with 0.7% sodium pentobarbital 24 h after PBS/ketamine treatment. Brains were rapidly removed and incubated in ice-cold artificial cerebrospinal fluid (ACSF) containing the following (in mM): 125 NaCl, 2.5 KCl, 1.3 NaH_2_PO_4_, 1.3 MgCl_2_, 2 CaCl_2_, 25 NaHCO_3_, 20 Glucose, bubbled with 95% O2 + 5% CO_2_, pH 7.4. S1 slices were cut at 400 μm using a VT-1200 Leica microslicer and allowed to recover in a submersion holding chamber with ACSF, bubbled with 95% O_2_ and 5% CO_2_ mixture at 25 °C – 28 °C for at least 1 h before recordings.

For whole-cell patch-clamp recording, brain slices were continuously perfused with oxygenated ACSF. The S1 region was visualized by contrast microscopy (Olympus, Japan). Whole-cell patch electrodes were pulled using P-97 (Sutter Instruments, United States) from borosilicate glass (1.5 mm outer diameter and 0.86 mm inner diameter; Sutter Instruments), and the resistances ranged from 3 to 5 MΩ. S1 layer II/III pyramidal neurons were recorded using an Axon 700B patch clamp amplifier (Axon Instruments, United States). The amplified signals were digitized at 10 kHz using a Digidata 1440A attached to pClampfit 10.1 (Axon Instruments, United States). Series and input resistances were continually monitored throughout all experiments. Data were not included when the series resistance changed by more than 20% during the experiment and was analyzed using Clamp 10.7.

For recording the spike frequency of neurons that respond to depolarizing current steps by current clamp, the intracellular solution contained (in mM): 140 K-gluconate, 11 EGTA, 2 MgCl_2_, 1 CaCl_2_, 10 HEPES, and 2 K_2_ATP; pH 7.3 (280–290 mOsm). A small current was injected to adjust the membrane potential to −70 mV. A family of 20 current steps were made in 10 pA increments, each for a duration of 3 s. For recording the mAHP currents, tetrodotoxin (TTX; 0.5 mM) and tetraethylammonium (TEA; 1 mM) were added to the ACSF. The patch pipettes were filled with a solution containing (in mM):125 KMeSO_4_, 5 KCl, 5 NaCl, 0.02 EGTA, 11 HEPES, 1 MgCl_2_, 10 Na_2_ phosphocreatine, 4 MgATP, and 0.3 NaGTP; pH 7.3 (280–290 mOsm). We voltage-clamped cells at −55 mV and applied 100 ms depolarization to 20 mV to elicit a robust Ca^2+^ action current followed by a return to −55 mV for 10 s (Bond et al., 2004). SK channel blocker apamin was used to examine the effect of SK channels on spike patterns and mAHP currents. Slices were incubated (for 10 min) and perfused within ACSF containing apamin (100 nM, MedChemExpress) or the vehicle (DMSO, 1 μL in 10 mL ACSF).

### 2.3 Brain removal and immunohistochemistry

Rats were deeply anesthetized with an intraperitoneal injection of 0.7% sodium pentobarbital (200 mg/kg) after PBS/ketamine treatment. Once unresponsive to noxious stimuli, rats were intracardially perfused with normal saline and subsequently with the fixation solution (4% paraformaldehyde in 0.1 M phosphate buffer, pH 7.4).

Brains were dissected and immersion-fixed in 4% PFA/PBS for 4–6 h at 4 °C and equilibrated in 30% sucrose. Coronal sections (30 μm) containing the primary somatosensory cortex were cut with a Leica CM1950 cryostat (Wetzlar, Germany), and every 5th section was immunostained. The blocking solution contains 5% bovine serum albumin (BSA) and 0.5% Triton X-100 for 2 h at 37 °C. Primary rabbit monoclonal antibody against cleaved caspase-3 (CC3, 1:400, Cell Signaling Technology, Cat# 9661) was applied overnight in 0.3% BSA at 4 °C. Washed 3 times in PBS, sections were stained for 2 h at room temperature (RT) in Alexa Fluor-conjugated (488 or 568 nm) secondary antibody at 1:500 (Thermo Fisher Scientific). Nuclei were labelled with DAPI (1:10,000, Sigma, Cat# D9542) for 15 min at RT. To quantify CC3^+^ cells, images were acquired with a Zeiss Pascal confocal microscope (Jena, Germany) and a ×20 Fluor objective (N.A. = 0.5) with a Z-step of 10 μm. Ten S1-containing sections, covering the entire S1 region, were imaged per pup. Images were analyzed by an experimenter blinded to the grouping using ImageProPlus software (Media-Cybernetics, Rockville, MD, United States).

### 2.4 Protein preparations and immunoblot analysis

The rats were deeply anesthetized with an intraperitoneal injection of 0.7% sodium pentobarbital 24 h after PBS/ketamine treatment. After the rats were decapitated, the regions encompassing the bilateral S1 were dissected and preserved in liquid nitrogen. To extract the total protein, S1 tissues were homogenized in ice-cold RIPA lysis buffer (Beyotime Biotechnology) supplemented with freshly added protease inhibitor cocktail tablets (Roche), lysed on ice for 30 min, and centrifuged at 4 °C for 10 min. Membrane protein fractions of S1 tissues were obtained with a Mem-PERTM Plus Membrane Protein Extraction Kit (Thermo Scientific). Protein samples were heated at 95 °C for 10 min in the loading buffer, and equal amounts were loaded onto SDS-PAGE. Western blots were accomplished according to standard protocols. The following primary antibodies were used: SK1 (1:500, Alomone Labs, Cat# APC-039), SK2 (1:500, Alomone Labs, Cat# APC-028), SK3 (1:500, Alomone Labs, Cat# APC-025), β-actin (1:1,000, Millipore, Cat# A1978), and pan-cadherin (1:1,000, Sigma-Aldrich, Cat# SAB4500001). HRP-conjugated secondary goat anti-rabbit or goat anti-mouse antibodies (Jackson ImmunoResearch) were used at 1:10,000. Blots were performed using ECL chemiluminescence substrate (Invitrogen). Results were analyzed using ImageJ software (NIH Image).

### 2.5 Co-immunoprecipitation

Co-immunoprecipitation was performed using the EZ View Red Protein G Affinity Gel (Sigma-Aldrich). Proteins were extracted from the bilateral S1 24 h after PBS/ketamine administration using an NP-40 Lysis Buffer (Beyotime Biotechnology) and supplemented with protease inhibitor cocktail tablets (Roche). Briefly, following centrifugation at 8,200 *g* at 4 °C for 10 min, the supernatant was incubated with anti-SK2 or anti-IgG (for a negative control) antibodies overnight at 4 °C, then carefully transferred into tubes prepared with prewashed EZview Red Protein G Affinity Gel beads overnight at 4 °C. After washing, bound proteins were eluted with SDS-sample buffer, separated by SDS-PAGE on a 6% gradient gel, and transferred to a PVDF membrane. The following Western blot parts were carried out as described above.

### 2.6 Reverse transcription-quantitative polymerase chain reaction

Total RNAs were extracted from the S1 using a High Pure RNA Tissue Kit (Roche) according to the protocols from manufacturer. Real-time quantitative PCR was performed to analyze levels of the SK1-3 subtypes transcripts using a StepOne Real-Time PCR System (ABI). Primer 5.0 software (Premier, Palo Alto, CA) was used to design the primers for SK1-3 genes, and the specificity of the primers was confirmed by a BLAST search (http://www.ncbi.nlm.nih.gov/blast/Blast.cgi). GAPDH served as an endogenous control gene. The following primers were used for qRT-PCR: SK1: 5′-TCA​TTC​GCC​CTG​AAA​TGC​CT-3’ (forward), 5′-CAGCGAGAT- CAGGGACACTC-3’ (reverse); SK2: 5′-TTC​TAA​CAA​CCT​GGC​GCT​CT-3’ (forward), 5′-GCT​TGC​GCT​TCT​CAA​ACA​GG-3’ (reverse); SK3: 5′-ACCATCATC- CTGCTTGGTTTGA-3’ (forward), 5′-TGC​CGT​CCA​GAA​GAA​CTT​GTA-3’ (reverse); and GAPDH: 5′-GTC​TTC​ACC​ACC​ATG​GAG​AA-3’ (forward), 5-TAAGCAGTT- GGTGGTGCAG-3’ (reverse). All reactions were performed in triplicate, and the amount of mRNA was calculated by absolute quantitation.

### 2.7 *In vivo* stereotaxic injections and transfection range confirmation

P0 pups were anesthetized by hypothermia for 2–4 min until movement ceased. Adeno-associated viruses (AAVs, type 2/9, packaged by Obio Technology, Shanghai, China) were injected bilaterally into S1 (0.1 μL/min, 1 μL per hemisphere), as previously described (Wang et al., 2017). Pups were returned to dams after fully awaking from anesthesia.

To overexpress SK2, AAV expressing SK2 fused with enhanced green fluorescent protein (EGFP) under the control of hSyn promoter (pAAV-hSyn-EGFP-P2A-Kcnn2-3xFLAG-WPRE, AAV-SK2 for short, 1.11 × 10^13^ vector genomes/ml) was constructed, and pAAV-SYN-EGFP-P2A- MCS-3FLAG (AAV-EGFP, 1.54 × 10^13^ vector genomes/ml) was employed as a negative control. To knock down the SK2, AAV expressing SK2-shRNA fused with EGFP (pAAV-CBG-EGFP-3xFLAG-WPRE-H1-shRNA (Kcnn2), shRNA-SK2 for short, 6.62 × 10^12^ vector genomes/ml) was constructed, and pAKD-CMV-bGlobin- eGFP-H1-shRNA-NC, (shRNA-Ctrl, 2.19 × 10^13^ vector genomes/ml) was employed as a negative control.

The expression of viruses was checked at P8. After the perfusion with 0.1 M PBS, and the following 4% PFA, the brains were removed and post-fixed in 4% PFA/PBS for 4–6 h at RT, and equilibrated in 30% sucrose. Coronal brain sections were obtained using a freezing microtome (Leica) at 30 μm. The injection site and transfection range were confirmed using TissueFAXS Plus ST (TissueGnostics GmbH, Vienna, Austria).

### 2.8 Statistical analysis

Statistical analyses were performed using Graph Pad Prism 9 (Graph Pad Software, La Jolla, CA, United States). As specified in figure legends, data distribution was tested for normality using the D’Agostino and Pearson test. For normally distributed data, unpaired two-tailed Student’s t-tests or two-way ANOVA followed by Tukey’s multiple comparisons test were used. For non-normally distributed data, the Mann-Whitney U test was employed. Numbers of cells and rats are reported in the figure legends. The experiments reported here were repeated independently at least three times, and at least three mice from two or more litters were used for each experimental condition. No statistical methods were used to predetermine sample sizes. Data were analyzed blindly for all experiments except electrophysiology, where a minimum of 50% underwent blinded analysis. Data were not collected in a blinded fashion. Data are shown as mean ± SEM, and the accepted value for significance was P < 0.05.

## 3 Results

### 3.1 Compensatory reduction in neuronal apoptosis and elevated neuronal excitability in S1 at 24 h post-ketamine anesthesia

**FIGURE 1:**
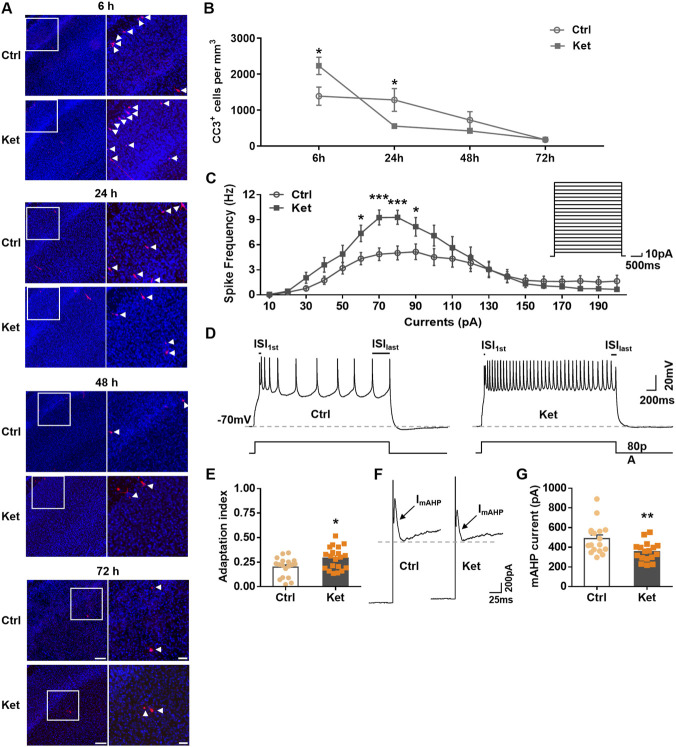
At 24 h post-ketamine anesthesia, neuronal apoptosis in S1 showed a compensatory reduction, and neuronal excitability increased. **(A,B)** The dynamic changes of neuronal apoptosis in neonatal rat S1 following a single exposure to ketamine. **(A)** Representative confocal images of S1 sections labelled for CC3 (red) and DAPI (blue), conditions as indicated; scale bar is 150 μm. Zoomed images of boxed regions are presented to the right of each panel; scale bar is 50 μm; CC3^+^ cells are indicated by white triangles. **(B)** Quantitation of the number of CC3^+^ cell per mm^3^ S1, treatment conditions as indicated. 3–6 rats were used per condition. **(C)** Plots of spike frequency versus current injected for layer II/III pyramidal neurons in S1. The neuronal spike frequency significantly increased at 24 h post-ketamine anesthesia. Inset, stimulus protocol. Ctrl, 25 neurons from 9 rats; Ket, 22 neurons from 8 rats. **(D,E)** The spike-frequency adaptation significantly decreased at 24 h post-ketamine anesthesia. **(D)** Representative responses to 3 s, 80 pA injected current. Bottom panel, stimulus protocol. ISI_1st_ and ISI_last_ refer to the first and the last inter-spike intervals (ISI), respectively. **(E)** Summary data showing the index for spike-frequency adaptation of the 3 s, 80 pA current-evoked spikes (Adaptation index = ISI_1st_/ISI_last_). Ctrl, 18 neurons from 9 rats; Ket, 20 neurons from 9 rats. **(F,G)** Representative traces **(F)** and column chart **(G)** showing the amplitude of mAHP current in each condition. Ctrl, 17 neurons from 6 rats; Ket, 17 neurons from 8 rats. *P < 0.05, **P < 0.01, ***P < 0.001. Data were analyzed using the Mann-Whitney U test for **(G)** and unpaired two-tailed Student’s t-tests for the other panels. Ctrl, control (PBS); Ket, ketamine. Data are shown as the mean ± SEM.

Although a single exposure to anesthetic has been shown to induce significant neuronal apoptosis in the developing brain (Chen et al., 2016; Wang et al., 2017), several studies report no long-term differences in neuronal density (Brambrink et al., 2010; Chen et al., 2016). To investigate whether compensatory mechanisms regulate ketamine-induced neuronal apoptosis, we first quantified apoptotic cells in S1 at various time points after ketamine administration, using cleaved caspase-3 (CC3) as an apoptotic marker. P7 rats received a single intraperitoneal injection of ketamine (60 mg/kg). Consistent with our previous report (Wang et al., 2017), ketamine significantly increased the number of cleaved caspase-3 positive (CC3^+^) cells in S1 at 6 h (h) compared with controls (6 h, Ctrl: 1386.11 ± 253.63/mm^3^ vs. Ket: 2229.07 ± 239.78/mm^3^, *P* = 0.0247, Figures 1A,B). However, by 24 h post-injection (P8), the number of CC3^+^ cells in S1 was significantly reduced in ketamine-treated animals (24 h, Ctrl: 1281.35 ± 316.07/mm^3^ vs. Ket: 554.24 ± 59.43/mm^3^, *P* = 0.0417, Figures 1A,B). At 48 h (P9), the number of CC3^+^ cells in S1 remained lower in the ketamine group, though not significantly (48 h, Ctrl: 722.91 ± 231.06/mm^3^ vs. Ket: 421.64 ± 55.48/mm^3^, *P* = 0.1332, Figures 1A,B). By 72 h (P10), the number of CC3^+^ cells was low in both groups and did not differ significantly (72 h, Ctrl: 173.51 ± 20.53/mm^3^ vs. Ket: 180.03 ± 15.60/mm^3^, *P* = 0.8009, Figures 1A,B). These results suggest that a single exposure to ketamine induces transient neuronal apoptosis in S1 of neonatal rats, followed by a compensatory inhibition of subsequent physiological apoptosis.

Given the crucial role of neuronal activity in cortical neuron survival during development (Blanquie et al., 2017a; Blanquie et al., 2017b; Priya et al., 2018), we next examined whether reduced neuronal apoptosis at 24 h post-ketamine resulted from altered neuronal activity. The neuronal spike frequency and pattern dynamically regulate signal transmission to projecting neurons (Destexhe and Marder, 2004), and are critical for cell survival during brain development (Wong Fong Sang et al., 2021). Whole-cell recordings from layer II/III pyramidal neurons in S1 revealed that ketamine-treated pups exhibited significantly elevated spike frequencies in response to depolarizing current steps compared with controls (Ctrl vs. Ket: 60 pA, *P* = 0.0161; 70 pA, *P* = 0.0004; 80 pA, *P* = 0.0008; 90 pA, *P* = 0.0432; Figure 1C). In addition, spike patterns were altered concomitant with increased frequency. Spike-frequency adaptation (SFA), reflected by the prolongation of inter-spike intervals (ISI) during sustained firing, was quantified using an adaptation index defined as the ratio of the first to the last ISI (ISI_1_/ISI_last_) (Figure 1D). Values closer to 0 indicate stronger adaptation, whereas values closer to 1 indicate weaker adaptation (Ha and Cheong, 2017). Based on the 80 pA current—which induced nearly maximal spikes in both groups—we found that the adaptation index was significantly higher in ketamine-treated rats (Ctrl: 0.20 ± 0.02 vs. Ket: 0.29 ± 0.03, *P* = 0.0122, Figure 1E), indicating reduced SFA.

The mAHP plays key roles in controlling the neuronal spike frequency and pattern, with decreased mAHP current leading to higher spike frequency and weaker adaptation (Gill and Hansel, 2020; Power and Sah, 2008; Yamamoto et al., 2019), so we further asked whether the changes in neuronal spike frequency and adaptation resulted from reduced mAHP currents at 24 h post-ketamine. Recordings from layer II/III pyramidal neurons showed that the mAHP current amplitude was significantly smaller in ketamine-treated rats than in controls (Ctrl: 487.33 ± 38.00 pA vs. Ket: 355.33 ± 23.49 pA, *P* = 0.0054, Figures 1F,G).

### 3.2 Apamin-sensitive SK channels mediate altered neuronal excitability at 24 h post-ketamine

**FIGURE 2:**
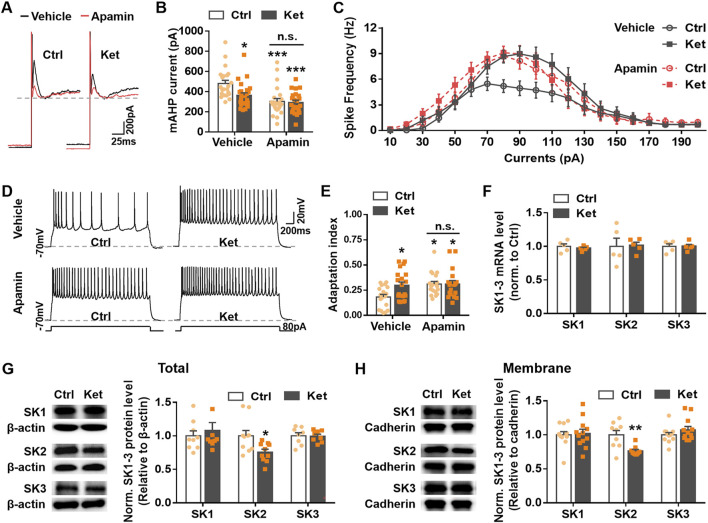
Apamin-sensitive SK2 channel-mediated mAHP currents may be linked to changes in neuronal spike frequency and adaptation at 24 h post-ketamine anesthesia. **(A,B)** Representative traces **(A)** and amplitudes **(B)** of the mAHP currents, treatment conditions as indicated. S1 slices from control (Ctrl) and ketamine-treated (Ket) rats were separately incubated and perfused with apamin (100 nM) or its vehicle. 22–25 neurons from 7–10 rats were used per condition. **(C)** Plots of spike frequency vs. current injected for layer II/III pyramidal neurons of S1. The significant differences in the spike frequency between the Ctrl and Ket groups (Ctrl: Vehicle vs. Ket: Vehicle, 80 pA, 100 pA and 110 pA, *P* < 0.01; 90 pA, *P* < 0.001) were eliminated after apamin treatment (Ctrl: Apamin vs. Ket: Apamin, *P* > 0.05). 20–22 neurons from 7–9 rats were recorded per condition. **(D,E)** Spikes in S1 layer II/III pyramidal neurons evoked for 3 s, 80 pA current injection **(D)**, and the adaptation index **(E)** was obtained by the algorithm mentioned above. 18–20 neurons from 7–9 rats were recorded per condition. **(F)** Quantitative analysis of SK1-3 mRNA in S1 of P8 rats. 5 rats were used per condition. *P* > 0.05. **(G,H)** Immunoblots and quantitative analysis of total **(G)** and membrane-bound **(H)** SK1-3 levels in S1 of ketamine-treated rats, normalized to corresponding levels in control rats. 8–12 rats were used per condition. *P < 0.05, **P < 0.01, ***P < 0.001; *n.s*., not significant. Data were analyzed using the Mann-Whitney U test for **(F,H)** and unpaired two-tailed Student’s t-tests for the other panels. Data are shown as the mean ± SEM.

Previous studies indicate that mAHP currents in cortical pyramidal neurons are primarily mediated by SK channels (Villalobos et al., 2004). To determine whether decreased SK channel activity contributed to the reduced mAHP currents observed in ketamine-treated rats, we perfused S1 slices with apamin (100 nM), a selective SK channel blocker (Faber et al., 2005; Köhler et al., 1996). Apamin significantly reduced mAHP current amplitude in control neurons (Ctrl, vehicle: 481.91 ± 31.13 pA vs. apamin: 303.75 ± 28.83 pA, *P* < 0.0001) but had no significant effect in neurons from ketamine-treated pups (Ket, vehicle: 364.02 ± 24.52 pA vs. apamin: 292.42 ± 22.30 pA, *P* = 0.2322, Figures 2A,B). After apamin application, mAHP amplitudes did not differ between groups (Apamin, Ctrl: 303.75 ± 28.83 pA vs. Ket: 292.42 ± 22.30 pA, *P* = 0.9907, Figures 2A,B), suggesting that the apamin-sensitive, SK-mediated component of the mAHP current reduces at 24 h post-ketamine anesthesia.

We next examined whether this reduction in SK-mediated mAHP current underlies the observed changes in spike frequency and adaptation. Apamin significantly increased spike frequency in control rats (80 pA: P = 0.0035; 90 pA: P *=* 0.0108) but had no effect in ketamine-treated animals (*P* > 0.05; Figure 2C). After apamin, spike frequencies did not differ between groups. Similarly, apamin significantly increased the adaptation index in controls (Ctrl, vehicle: 0.18 ± 0.03 vs. apamin: 0.31 ± 0.03, *P =* 0.0163) but not in ketamine-treated rats (Ket, vehicle: 0.30 ± 0.03 vs. apamin: 0.31 ± 0.03, *P =* 0.9911) (Figures 2D,E). The difference in adaptation index between groups was abolished by apamin treatment (Apamin, Ctrl: 0.31 ± 0.03 vs. Ket: 0.31 ± 0.03, *P* > 0.9999, Figures 2D,E). These results indicate that increased spike frequency and reduced SFA in layer II/III pyramidal neurons 24 h after ketamine injection are mediated by reduced apamin-sensitive SK currents.

We then asked whether decreased SK current resulted from reduced SK channel expression. Quantitative PCR revealed no significant differences in mRNA levels of SK1, SK2, or SK3 channel isoforms between groups (*P* > 0.05; Figure 2F). However, Western blot analysis showed that both total and membrane-bound SK2 protein levels were significantly lower in ketamine-treated rats at 24 h post-anesthesia (total: 0.75 ± 0.04, *P* = 0.0156, Figure 2G; membrane: 0.76 ± 0.02, *P* = 0.0078; Figure 2H), whereas SK1 and SK3 levels were unchanged (Total, SK1: 1.08 ± 0.12, *P* = 0.5697; SK3: 0.99 ± 0.03, *P* = 0.9162; Figure 2G) (Membrane, SK1: 1.02 ± 0.06, *P* = >0.9999; SK3: 1.07 ± 0.05, *P* = 0.4037; Figure 2H).

### 3.3 SK2 overexpression reverses neuronal hyperactivity and apoptosis reduction at 24 h post-ketamine anesthesia

**FIGURE 3:**
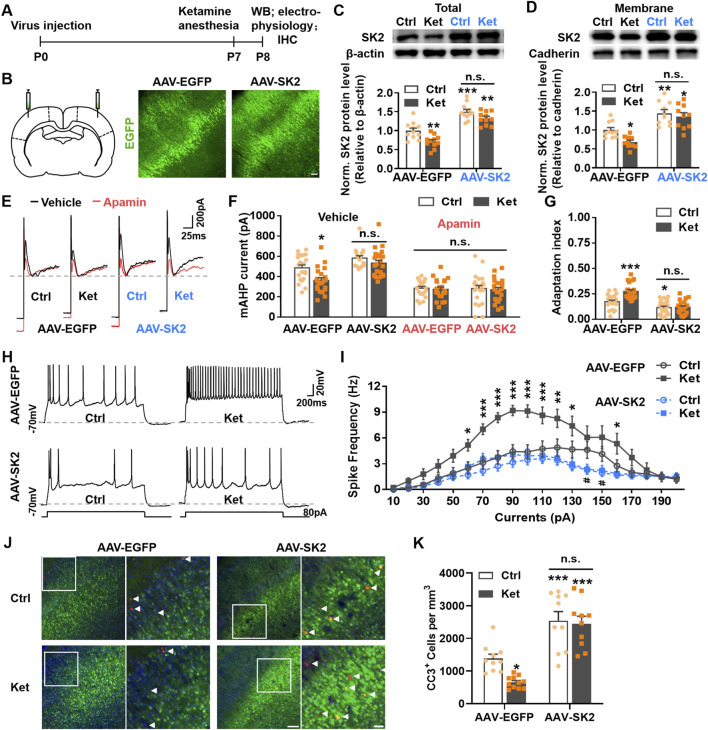
Overexpression of SK2 reversed the increase in spike frequency and prevented the reduction in apoptosis at 24 h post-anesthesia. **(A)** Schematic of the experimental procedure. **(B)** Left panel: Schematic drawing showing the location of the AAV-injection, AAV-SK2 (pAAV-hSyn-EGFP-P2A-Kcnn2-3xFLAG-WPRE) or AAV-EGFP (pAAV-SYN-EGFP-P2A-MCS-3FLAG) were injected bilaterally into S1 at P0; Right two panels: representative images showing EGFP positive neurons (indicated the virus transfected neurons) in S1 of P8 rats. The scale bar is 100 μm. **(C,D)** Immunoblots and quantitation of SK2 levels from the total **(C)** or membrane **(D)** lysates in S1. Conditions as indicated. 10–11 rats were used per condition. **(E,F)** Representative traces **(E)** and amplitudes **(F)** of the mAHP currents, treatment conditions as indicated. AAV-EGFP- and AAV-SK2-treated rats had received PBS (Ctrl) or ketamine (Ket) at P7, and the acute brain slices containing S1 were incubated and perfused with apamin or its vehicle 24 h later. 18–24 neurons from 4 rats were used per condition. **(G,H)** A depolarizing current of 80 pA was injected for 3 s to induce neuronal spikes in S1 **(H)**, and the adaptation index of the spikes was analyzed **(G)** in conditions as indicated. 20–24 neurons from 4–5 rats were used per condition. **(I)** Plots of spike frequency evoked by graded current injections. AAV-EGFP: Ctrl vs. Ket, *P < 0.05, **P < 0.01, ***P < 0.001; Ctrl: AAV-EGFP vs. AAV-SK2, ^#^ *P* < 0.05; using two-way ANOVA followed by Tukey’s multiple comparison test. 20–29 neurons from 4 rats were used per condition. **(J)** Representative confocal images of S1 sections labelled with CC3 (red), EGFP (green) and DAPI (blue), conditions as indicated; scale bar is 150 μm. Zoomed images of boxed regions are presented to the right of each panel; scale bar is 50 μm; CC3+ cells are indicated by white triangles. **(K)** Quantitation of the number of CC3^+^ cell per mm^3^ S1, treatment conditions as indicated. 5–8 rats were used per condition. *P < 0.05, **P < 0.01, ***P < 0.001; *n.s*., not significant; using two-way ANOVA followed by Tukey’s multiple comparison tests. Data are shown as the mean ± SEM.

To determine whether reduced SK2 protein levels contributed to increased neuronal activity and the consequent decrease in neuronal apoptosis at 24 h post-ketamine anesthesia, we bilaterally injected adeno-associated virus (AAV) expressing SK2 into S1 at postnatal day 0 (P0) to overexpress SK2 channels. Rats underwent ketamine anesthesia at P7, and AAV expression was confirmed at P8 (Figures 3A,B). Both total and surface-expressed SK2 channels in S1 were significantly upregulated in AAV-SK2-expressing control (Total, AAV-EGFP: 1.00 ± 0.07 vs. AAV-SK2: 1.50 ± 0.06, *P* < 0.0001; Membrane, AAV-EGFP: 1.00 ± 0.07 vs. AAV-SK2: 1.44 ± 0.10, *P* = 0.0025) and ketamine-treated pups (Total, AAV-EGFP: 0.71 ± 0.05 vs. AAV-SK2: 1.33 ± 0.05, *P* < 0.0001; Membrane, AAV-EGFP: 0.69 ± 0.05 vs. AAV-SK2: 1.36 ± 0.10, *P* < 0.0001) (Figures 3C,D). Notably, SK2 overexpression abrogated the ketamine-induced reduction in SK2 levels (Figures 3C,D). Consistent with these changes, the ketamine-induced decrease in the amplitude of mAHP currents was reversed by AAV-SK2 expression. Recordings from layer II/III pyramidal neurons in AAV-SK2-expressing S1 revealed that mAHP current amplitudes in the ketamine group were restored to control levels (AAV-SK2, Ctrl: 583.32 ± 22.77 pA vs. Ket: 531.95 ± 27.24 pA, *P* = 0.8600, Figures 3E,F). This SK2 overexpression-mediated increase in mAHP current amplitude was fully blocked by apamin (Figures 3E,F).

Given that SK2 overexpression restored both SK2 channel levels and mAHP current amplitudes in ketamine-treated rats, we next assessed its effects on neuronal spike frequency and spike frequency adaptation at 24 h post-ketamine. AAV-SK2 expression significantly attenuated the spike frequency adaptation index. The ketamine-induced increase in adaptation index (AAV-EGFP, Ctrl: 0.17 ± 0.02 vs. Ket: 0.27 ± 0.02, *P* < 0.0001) was abolished by SK2 overexpression (AAV-SK2, Ctrl: 0.12 ± 0.01 vs. Ket: 0.12 ± 0.01, P = 0.9975) (Figures 3G,H). Similarly, AAV-SK2 injection reduced neuronal spike frequency and fully reversed the increase in spike frequency of ketamine-treated rats (Figure 3I).

Having established that SK2 overexpression suppresses pyramidal neuron hyperactivity in S1, we investigated whether it counteracts the compensatory reduction in neuronal apoptosis at 24 h post-ketamine. SK2 overexpression significantly increased neuronal apoptosis in both control (AAV-EGFP: 1380.82 ± 133.45/mm^3^ vs. AAV-SK2: 2541.51 ± 278.59/mm^3^, *P* = 0.0009) and ketamine-treated pups (AAV-EGFP: 652.01 ± 57.83/mm^3^ vs. AAV-SK2: 2447.58 ± 230.44/mm^3^, *P* < 0.0001). Consequently, the number of CC3^+^ cells did not differ between control and ketamine groups in AAV-SK2-expressing rats (Ctrl: 2541.51 ± 278.59/mm^3^ vs. Ket: 2447.58 ± 230.44/mm^3^, P = 0.9861, Figures 3J,K). These results implicate SK2 channels in regulating the compensatory apoptosis reduction post-ketamine, likely via modulation of local network activity.

### 3.4 SK2 knockdown mimics ketamine effects on neuronal activity and apoptosis in control rats at 24 h

**FIGURE 4:**
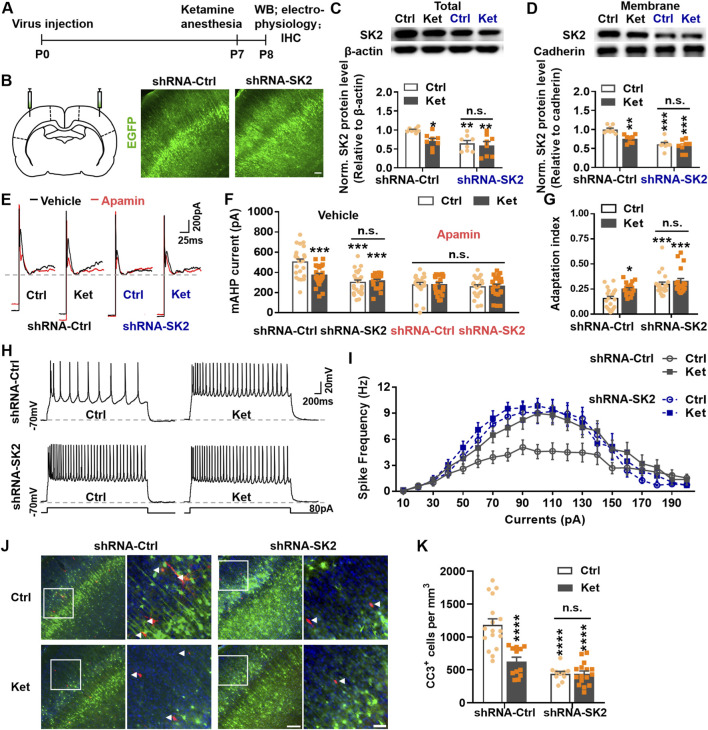
SK2 downregulation recapitulated neuronal hyperexcitability and further reduced the number of apoptotic neurons at 24 h post-anesthesia. **(A)** Schematic of the experimental procedure. **(B)** Left panel: Schematic drawing showing the location of the AAV injection, shRNA-SK2 [pAAV-CBG-EGFP-3xFLAG-WPRE-H1-shRNA (Kcnn2)] or shRNA-Ctrl (pAKD-CMV-bGlobin-eGFP-H1-shRNA-NC) were injected bilaterally into S1 at P0; Right two panels: representative images showing EGFP positive neurons (the virus transfected neurons) in S1 of P8 rats. The scale bar is 100 μm. **(C,D)** Immunoblots and quantitation of SK2 from the total **(C)** or membrane **(D)** lysates of S1 on P8. Conditions as indicated. 8 rats were used per condition. **(E,F)** Representative traces **(E)** and the amplitude **(F)** of the mAHP currents, treatment conditions as indicated. 20–23 neurons from 4 rats were used per condition. **(G,H)** A depolarizing current of 80 pA was injected for 3 s to induce neuronal spikes in S1 **(H)**, and the adaptation index of the spikes was analyzed **(G)** using the mentioned methods. 18–20 neurons from 4–5 rats were used per condition. **(I)** Plots of spike frequency evoked by stepping current injections. No significant difference was found between Ctrl and Ket groups following shRNA-SK2 virus transfection (*P* > 0.05). (shRNA-Ctrl: Ctrl vs. Ket): 80 pA, 90 pA, and 120 pA, *P* < 0.05; 100 pA and 110 pA, *P* < 0.01; using two-way ANOVA followed by Tukey’s multiple comparison test. 20–21 neurons from 4–5 rats were used per condition. **(J)** Representative confocal images of S1 sections labelled with CC3 (red), EGFP (green) and DAPI (blue), conditions as indicated; scale bar is 150 μm. Zoomed images of boxed regions are presented to the right of each panel; scale bar is 50 μm; CC3^+^ cells are indicated by white triangles. **(K)** Quantitation of the number of CC3^+^ cell per mm^3^ S1, treatment conditions as indicated. 3–4 rats were used per condition. *P < 0.05, **P < 0.01, ***P < 0.001, ****P < 0.0001; *n.s*., not significant; using two-way ANOVA followed by Tukey’s multiple comparison test. Data are shown as the mean ± SEM.

We further tested whether knocking down SK2 expression in control rats could mimic the effects of ketamine. To examine this, we used an AAV-shRNA gene silencing approach to downregulatle the expression of SK2 channels. Using a protocol similar to the above experiment, the AAV-shRNA-Ctrl and AAV-shRNA-SK2 were microinjected into the S1, and were well expressed (Figures 4A,B). In shRNA-Ctrl groups, we observed reduced total (Ctrl: 1.00 ± 0.02 vs. Ket: 0.71 ± 0.07, *P* = 0.0417, Figure 4C) and membrane-bound (Ctrl: 1.00 ± 0.04 vs. Ket: 0.75 ± 0.04, *P* = 0.0037, Figure 4D) SK2 levels in ketamine-treated rats versus controls. However, shRNA-SK2 significantly reduced SK2 expression in control rats, eliminating differences between control and ketamine groups (Total, Ctrl: 0.65 ± 0.08 vs. Ket: 0.60 ± 0.10, *P* = 0.9482, Figure 4C) (Membrane, Ctrl: 0.61 ± 0.05 vs. Ket: 0.56 ± 0.06, *P* = 0.8794, Figure 4D). Accordingly, shRNA-SK2 reduced apamin-sensitive mAHP current amplitudes in control rats, and no difference was observed between groups (shRNA-SK2, Ctrl: 300.68 ± 22.47 pA vs. Ket: 315.30 ± 16.19 pA, *P* = 0.9998, Figures 4E,F).

Recordings from shRNA-expressing pyramidal neurons revealed that SK2 knockdown in control rats recapitulated ketamine’s effects: the adaptation index increased in shRNA-Ctrl ketamine-treated rats (Ctrl: 0.16 ± 0.02 vs. Ket: 0.25 ± 0.01, *P* = 0.0301), while shRNA-SK2 expression elevated the adaptation index in control rats, erasing group differences (Ctrl: 0.30 ± 0.02 vs. Ket: 0.33 ± 0.03, *P* = 0.7887) (Figures 4G,H). Similarly, shRNA-SK2 enhanced spike frequencies in control rats, aligning them with ketamine-treated pups (Figure 4I).

Finally, SK2 knockdown in control rat S1 reduced neuronal apoptosis to levels indistinguishable from ketamine-treated rats (shRNA-SK2, Ctrl: 440.43 ± 35.73/mm^3^ vs. Ket: 439.91 ± 43.02/mm^3^, *P* > 0.9999). In contrast, shRNA-Ctrl ketamine-treated rats exhibited fewer CC3^+^ cells than controls (Ctrl: 1183.60 ± 91.54/mm^3^ vs. Ket: 628.10 ± 63.48/mm^3^; *P* < 0.0001; Figures 4J,K). SK2 knockdown did not significantly alter apoptosis in ketamine-treated rats (shRNA-Ctrl: 628.10 ± 63.48/mm^3^ vs. shRNA-SK2: 439.91 ± 43.02/mm^3^; *P* = 0.2338; Figures 4J,K).

Collectively, ketamine administration reduced total and surface SK2 channel levels at 24 h, diminishing SK2-mediated mAHP currents and increasing spike frequency. SK2 overexpression in S1 reversed ketamine-induced neuronal hyperactivity and elevated apoptosis. Conversely, SK2 knockdown in control rats mimicked ketamine’s effects on neuronal activity and apoptosis but did not exacerbate these phenotypes in ketamine-treated rats.

### 3.5 Ubiquitin-proteasome-mediated degradation underlies SK2 downregulation

**FIGURE 5:**
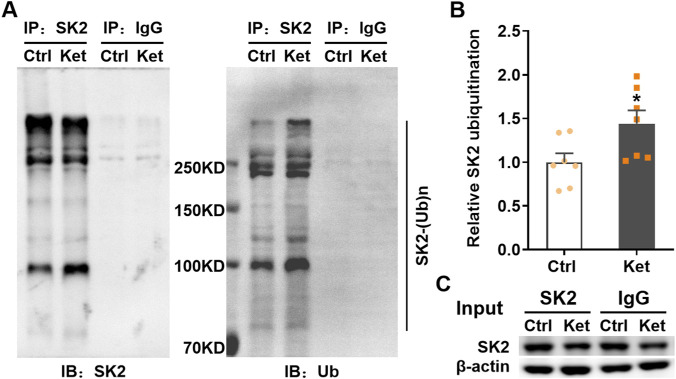
The ubiquitination level of SK2 channels in S1 significantly increased at 24 h post-ketamine anesthesia. **(A)** Immunoprecipitation was performed with anti-SK2 antibodies or control IgG antibodies in PBS (Ctrl) and ketamine (Ket) treated rats, and western blots were labelled with anti-SK2 (left panel) or anti-ubiquitin (Ub) (right panel) antibodies. **(B)** The column graph shows a significant increase in ubiquitination of SK2 in S1 at 24 h after ketamine exposure. *P < 0.05, unpaired two-tailed Student’s t-tests. 7 rats were used per condition. **(C)** Input protein levels examined by Western blot probed with SK2 and β-actin antibodies, conditions as indicated. Data are shown as the mean ± SEM.

We next investigated the potential mechanism underlying the reduction in SK2 levels observed 24 h after ketamine administration. Since SK2 mRNA levels were not significantly altered by ketamine anesthesia (Figure 2F), the decrease in both total and surface-expressed SK2 protein likely resulted from enhanced post-translational degradation. Previous studies indicate that SK2 protein degradation is predominantly mediated by the ubiquitin-proteasome system (UPS), which can be regulated in an activity-dependent manner (Müller et al., 2018; Sun et al., 2020; Sun et al., 2015). Therefore, we hypothesized that UPS might account for SK2 downregulation in S1 at 24 h post-ketamine, consequently affecting neuronal activity and apoptosis. To assess whether SK2 ubiquitination was altered, immunoprecipitation was performed using anti-SK2 antibodies, followed by Western blotting with anti-SK2 and anti-ubiquitin (Ub) antibodies. Despite lower overall SK2 protein levels in the S1 of ketamine-treated pups (Figure 5C), the relative level of ubiquitinated proteins co-precipitated with SK2 was significantly elevated compared to controls (1.44 ± 0.15, *P* = 0.0329; Figures 5A,B).

**FIGURE 6:**
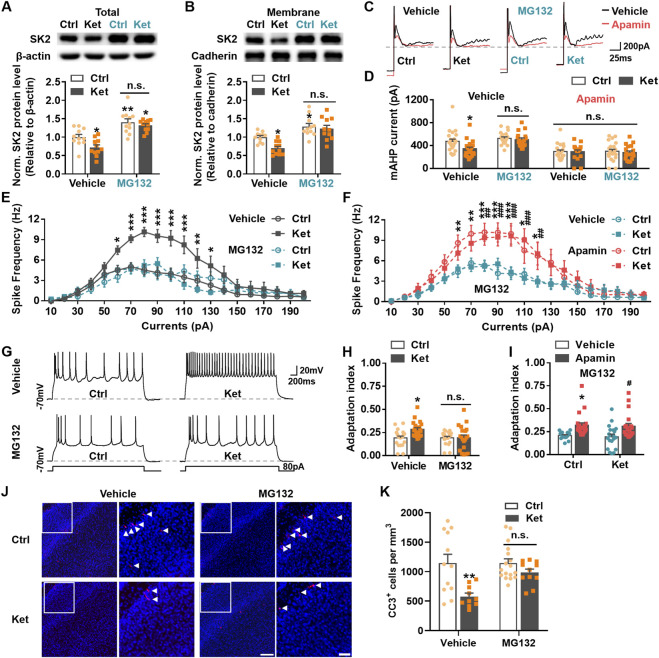
MG132 normalized neuronal spike frequency and abolished the compensatory reduction in apoptosis by restoring SK2 protein levels. **(A,B)** Immunoblots and quantitation of SK2 from the total **(A)** or membrane **(B)** lysates of S1 with conditions as indicated. 11 rats were used per condition. **(C,D)** Representative traces **(C)** and the amplitude **(D)** of the mAHP currents, treatment conditions as indicated. 18–22 neurons from 4–5 rats were used per condition. **(E)** Plots of spike frequency evoked by stepping current injections. MG132 pre-treatment eliminated the increase in neuronal spike frequency caused by ketamine. 20–26 neurons from 4–5 rats were used per condition. **(F)** Plots of spike frequency vs. current injections. The effects of MG132 on neuronal spike frequency were abolished by apamin. 21–22 neurons from 4 - 6 rats were used per condition. (Vehicle: Ctrl) vs. (Apamin: Ctrl), *P < 0.05, **P < 0.01, ***P < 0.001; (Vehicle: Ctrl) vs. (Apamin: Ket), ^##^ *P* < 0.01, ^*###*^ *P* < 0.001; Vehicle: Ctrl vs. Ket, and Apamin: Ctrl vs. Ket, *P* > 0.05; using two-way ANOVA followed by Tukey’s multiple comparison test. **(G,H)** A depolarizing current of 80 pA was injected for 3 s to induce neuronal spikes of S1 pyramidal neurons **(G)**, and the adaptation index of the spikes was analyzed **(H)** using the same algorithm mentioned. 20–21 neurons from 4–5 rats were used per condition. **(I)** The bar graph showed the effects of MG132 on spike adaptation were prevented by apamin. 17–22 neurons from 4–5 rats were used per condition. Ctrl: Vehicle vs. Apamin, *P < 0.05; Ket: Vehicle vs. Apamin, ^#^ *P* < 0.05; using two-way ANOVA followed by Tukey’s multiple comparison test. **(J)** Representative confocal images of S1 sections labelled with CC3 (red) and DAPI (blue), conditions as indicated; scale bar is 150 μm. Zoomed images of boxed regions are presented to the right of each panel; scale bar is 50 μm; CC3^+^ cells are indicated by white triangles. **(K)** Quantitation of the number of CC3^+^ cell per mm^*3*^ S1, treatment conditions as indicated. 4–7 rats were used per condition. *P < 0.05, **P < 0.01, ***P < 0.001; *n.s*., not significant; using two-way ANOVA followed by Tukey’s multiple comparison test. Data are shown as the mean ± SEM.

To determine if the increased ubiquitination facilitated SK2 degradation post-ketamine, MG132, a proteasome inhibitor of the UPS, was administered intraperitoneally 30 min prior to ketamine or PBS injection (Lu et al., 2017). MG132 significantly increased total SK2 levels in both control (Vehicle: 1.00 ± 0.07 vs. MG132: 1.40 ± 0.09, *P* = 0.0021) and ketamine-treated groups (Vehicle: 0.72 ± 0.07 vs. MG132: 1.32 ± 0.05, *P* < 0.0001), eliminating the difference between these groups (MG132, Ctrl: 1.40 ± 0.09 vs. Ket: 1.32 ± 0.05, *P* = 0.8446) (Figure 6A). Similarly, MG132 significantly increased surface-expressed SK2 levels in both control (Vehicle: 1.00 ± 0.04 vs. MG132: 1.29 ± 0.08, *P* = 0.030) and ketamine-treated rats (Vehicle: 0.70 ± 0.05 vs. MG132: 1.23 ± 0.09, *P* < 0.0001), also abolishing the intergroup difference (MG132, Ctrl: 1.29 ± 0.08 vs. Ket, 1.23 ± 0.09, *P* = 0.9307) (Figure 6B). These results demonstrate that MG132 reversed the ketamine-induced decrease in both total and surface-expressed SK2 in S1 at 24 h. Consistent with the alterations in SK2 levels, MG132 significantly eliminated the reduction in the amplitude of mAHP currents in ketamine-treated rats (Vehicle, Ctrl: 473.61 ± 39.46 pA vs. Ket: 344.25 ± 27.39 pA, *P* = 0.0441; MG132, Ctrl: 526.95 ± 24.23 pA vs. Ket; 502.88 ± 29.53 pA, *P* = 0.9995); these currents were fully blocked by 100 nM apamin (Figures 6C,D).

Given that MG132 eliminated the decrease in SK2 levels as well as the SK channel-mediated mAHP current component (Figures 6A–D), we examined whether it could also mitigate the ketamine-induced changes in neuronal activity and apoptosis in S1 at 24 h. Whole-cell recordings from pyramidal neurons showed that MG132 eliminated the ketamine-induced increase in spike frequency (*P* > 0.05; Figure 6E). MG132 also reversed the alteration in spike-frequency adaptation, as evidenced by the abolition of the increased adaptation index in ketamine-treated rats (Vehicle, Ctrl: 0.19 ± 0.02 vs. Ket: 0.28 ± 0.02, *P* = 0.0154; MG132, Ctrl: 0.19 ± 0.02 vs. Ket, 0.19 ± 0.03, *P* > 0.9999; Figures 6G,H). Furthermore, apamin (100 nM) significantly blocked MG132’s effects on neuronal spike frequency (Figure 6F) and spike-frequency adaptation (Figure 6I), suggesting that MG132 likely acted by restoring SK2 levels in S1. Additionally, MG132 significantly increased the number of CC3^+^ cells in the S1 of ketamine-treated rats (Ket, Vehicle: 578.24 ± 56.34/mm^3^ vs. MG132: 984.08 ± 60.43/mm^3^; *P* = 0.0364), with no significant difference remaining between the control and ketamine groups under MG132 treatment (MG132, Ctrl: 1144.61 ± 73.05/mm^3^ vs. Ket: 984.08 ± 60.43/mm^3^; *P* = 0.5992) (Figures 6J,K).

Together, these results demonstrate that increased SK2 ubiquitination and subsequent proteasomal degradation underlie the reduction in SK2 channels at 24 h post-ketamine anesthesia. This degradation pathway contributes to increased neuronal activity in S1 pyramidal neurons and decreased neuronal apoptosis.

## 4 Discussion

The developing central nervous system exhibits remarkable plasticity to counteract environmental stressors and maintain homeostatic balance. In this study, we uncovered a novel compensatory mechanism through which the developing somatosensory cortex (S1) restores neuronal cell number homeostasis following ketamine-induced acute apoptosis. Specifically, we demonstrate that an initial surge in apoptotic neurons at 6 h post-anesthesia is rapidly offset by a significant reduction in physiological apoptosis at 24 h, mediated by increased neuronal activity resulting from downregulation of SK2 potassium channels. Mechanistically, this SK2 downregulation is driven by enhanced ubiquitination and subsequent proteasomal degradation, adding a critical layer of post-translational regulation to our understanding of anesthetic-induced neuroplasticity.

Ketamine is a widely used pediatric general anesthetic. Our prior work demonstrated that a single ketamine dose induces dose-dependent cortical apoptosis, peaking during the physiological apoptosis window at postnatal days 5–7 (P5-P7) (Wang et al., 2017). Here, P7 rat pups received 60 mg/kg ketamine, a dose providing 2–3 h of surgical anesthesia without hypoxia (Wang et al., 2017). We observed significantly increased apoptotic neurons in S1 6 h post-injection, followed by a significant decrease by 24 h (P8) (Figures 1A,B). Although apoptotic counts in ketamine-treated pups remained lower than controls at P9 (48 h), this difference lacked statistical significance (Figures 1A,B). By P10 (72 h), apoptotic cell numbers declined to developmentally expected low levels (Ikonomidou et al., 1999; Wang et al., 2017), with no significant group differences (Figures 1A,B). These dynamics indicate rapid cortical compensation for anesthetic-induced apoptosis via suppression of physiological apoptosis.

All common general anesthetics induce neuronal apoptosis in developing animals, with the extent and duration dependent on anesthetic dose, anesthesia duration, or frequency (Alvarado et al., 2017; Li et al., 2025; Wang et al., 2022; Wang et al., 2017). Previous studies showed that single propofol exposure causes transient apoptosis and neuronal deficits in the hippocampal CA1 and prelimbic cortex of P9 rats, with normalization by P14; however, repeated exposure leads to persistent apoptosis and deficits (Chen et al., 2016). Similarly, single isoflurane exposure increases apoptosis in newborn dentate granule neurons, but counts recover by 60 days post-exposure (Jiang et al., 2016). These findings align with our observations of compensatory capacity following single anesthetic exposures. Crucially, prior studies may have overlooked transient physiological apoptosis reduction, as examinations occurred days post-anesthesia when apoptosis had normalized. Our data reveal that ketamine-induced S1 neuronal apoptosis peaks transiently at 6 h, then declines rapidly by 24 h to maintain neuronal homeostasis (Figures 1A,B). We acknowledge that postnatal dentate gyrus neurogenesis represents an alternative compensatory mechanism not excluded by our experimental design.

During early postnatal development, physiological apoptosis eliminates surplus cortical neurons to establish mature circuit architecture. While cell-intrinsic program, neurotrophic support, and pro-/anti-apoptotic factors modulate this process, neuronal activity plays a prominent role (Blanquie et al., 2017a; Dekkers et al., 2013). Indeed, increasing cortical network or intrinsic cellular activity via kainate injection, DREADD chemogenetic activation, or environmental enrichment (EE) reduces neuronal apoptosis (Blanquie et al., 2017a; Heck et al., 2008; Wang et al., 2017). Our study establishes that elevated activity in cortical pyramidal neurons underlies compensatory apoptosis reduction. We observed increased spike frequency and reduced spike-frequency adaptation in pyramidal neurons 24 h post-ketamine (Figures 1C–E), attributable to decreased SK2-mediated medium afterhyperpolarization (mAHP) currents (Figure 2). Crucially, SK2 overexpression suppressed neuronal activity and inhibited compensatory apoptosis reduction (Figure 3), while SK2 knockdown mimiced ketamine’s effects on neuronal activity and apoptosis in control rats but did not exacerbate these phenotypes in ketamine-treated rats (Figure 4). Beyond enhancing their own survival, elevated pyramidal neuron activity also promotes interneuron survival (Wong et al., 2018). Previous research showed that increased pyramidal neuron activity prevented interneuron apoptosis and increased their population, whereas decreased activity had the opposite effect (Wong et al., 2018). Although we did not directly measure interneuron activity, we speculate it may also increase. This is supported by studies showing postnatal cortex homeostatically modulates interneuron apoptosis through activity-dependent mechanisms, and interneuron loss reduces apoptosis cell-autonomously, likely due to acute activity increases (Denaxa et al., 2018a; Denaxa et al., 2018b).

We found that reduced SK2-mediated mAHP currents underlie the compensatory decrease in neuronal apoptosis 24 h post-ketamine anesthesia. SK channels, activated solely by intracellular Ca^2+^, mediate potassium efflux and membrane hyperpolarization (Adelman et al., 2012). SK1-3 channel isoforms are abundantly expressed in both the developing and mature brain (Gymnopoulos et al., 2014; Sailer et al., 2004). SK channel downregulation enhances spike frequency and attenuates spike frequency adaptation (SFA) by reducing mAHP, while overexpression has opposite effects (Adelman et al., 2012; Gill and Hansel, 2020; Power and Sah, 2008). As mAHP is predominantly SK-mediated, particularly in cortex (Villalobos et al., 2004), we investigated whether its reduction post-ketamine stemmed from diminished SK current. Application of the SK blocker apamin minimally affected mAHP amplitude in ketamine-treated pyramidal neurons but significantly reduced it in controls (Figures 2A,B). Post-apamin, mAHP amplitudes were comparable between groups (Figures 2A,B). Consistently, apamin eliminated differences in spike frequency (Figure 2C) and adaptation (Figures 2D,E) between control and ketamine groups. Apamin mimicked ketamine’s effects on increasing spike frequency and reducing adaptation at 24 h, suggesting these changes result from loss of apamin-sensitive (SK-mediated) mAHP. Quantification revealed significantly lower total and surface SK2 protein expression in the S1 of ketamine-treated rats versus controls (Figures 2G,H). To determine if SK2 downregulation mediates these neuronal activity and apoptosis changes, we modulated SK2 expression in rat S1. Both SK2 overexpression and knockdown abolished the differences in activity and apoptosis between control and ketamine-treated groups (Figures 3, 4). AAV-SK2 overexpression increased mAHP, reversed ketamine-induced changes in spike frequency/adaptation, and suppressed the compensatory apoptosis reduction (Figure 3). Conversely, shRNA-SK2 knockdown in controls replicated ketamine’s effects on spike frequency/adaptation and promoted reduced apoptosis (Figure 4).

While our findings establish that SK2 downregulation-mediated neuronal hyperexcitability drives the compensatory reduction in apoptosis following ketamine exposure, the precise downstream molecular mechanisms translating this elevated electrical activity into cell survival signals remain to be fully elucidated. It is well established that electrical activity influences cell survival or death primarily through the elevation of intracellular Ca^2+^. As reviewed by Blanquie et al. , the survival of developing neurons is strictly activity-dependent, where depolarization triggers calcium influx primarily through N-methyl-D-aspartate receptors (NMDARs) or voltage-gated calcium channels (VGCCs) (Blanquie et al., 2017a). Such calcium transients activate key survival kinases (e.g., CaMKII, PI3K/Akt, and ERK/MAPK pathways). These cascades subsequently induce the transcription and secretion of neurotrophic factors, particularly BDNF, and directly modulate the Bcl-2 family rheostat—upregulating anti-apoptotic proteins (Bcl-2, Bcl-xL) while sequestering or degrading pro-apoptotic effectors (Bax, Bad, and Caspase-9) (Dekkers et al., 2013; Schroer et al., 2023; Wong Fong Sang et al., 2021; Yan et al., 2024). Therefore, it is highly probable that the SK2-mediated increase of spike frequency in our model rescues neurons by re-engaging these calcium-dependent neuroprotective pathways.

Attenuation of SK2-mediated mAHP currents directly enhanced excitability in S1 pyramidal neurons of ketamine-treated rats. Prior *in vitro* studies show ketamine inhibits recombinant rat brain SK2 channels dose-dependently (Dreixler et al., 2000), and systemic low-dose ketamine (10 mg/kg, i.p.) produces rapid antidepressant effects by suppressing SK channel-mediated AHP in mPFC pyramidal neurons (Bambico et al., 2020). However, in the present study, the observed reduction in SK2-mediated mAHP currents is unlikely to result from direct blockade of SK2 channels by ketamine, as the measurements were performed 24 h after ketamine administration. We therefore explored alternative mechanisms underlying this decrease. Neonatal sevoflurane exposure impairs cognition in juvenile rats, associating with increased hippocampal SK2 surface expression and synaptic GluA2-lacking AMPAR incorporation (Ke et al., 2021; Yu et al., 2018); this SK2 upregulation was attributed to inhibited endocytosis (Ke et al., 2021). In contrast, our results revealed a significant decrease in both total and surface expression of SK2 channels in the S1 region of ketamine-treated rats (Figures 2G,H), with no change in SK2 mRNA levels (Figure 2F). These findings suggest that ketamine treatment may enhance the endocytosis and degradation of SK2 proteins. It remains an open question whether this SK2-mediated compensation is a ketamine-specific phenomenon or a more generalized response to NMDAR-targeted insults. Notably, a recent study demonstrated that nitrous oxide, another NMDAR antagonist, also triggers SK2 channel inhibition to modulate cortical excitability (Cichon et al., 2025). This suggests that SK2 downregulation may be a common homeostatic mechanism triggered by NMDAR blockade across different anesthetic agents. Given that many general anesthetics converge on NMDAR signaling or neuronal activity suppression, the surge in neuronal activity mediated by SK2 downregulation likely serves as a fundamental endogenous mechanism to counteract transient suppression during critical developmental windows.

Ubiquitination critically regulates protein degradation, including that of membrane ion channels and transporters by promoting their endocytosis and subsequent proteasomal or lysosomal degradation, or recycling back to the membrane (Lamothe and Zhang, 2016; Pohl and Dikic, 2019). Alterations in the ubiquitin-proteasome system following general anesthesia are documented. For instance, postnatal sevoflurane exposure reduces hippocampal PSD-95 levels via enhanced ubiquitination and degradation, impairing cognition in young mice—an effect reversed by proteasome inhibitor MG132 (Lu et al., 2017). Similarly, isoflurane inhibits GluA1 ubiquitination, increasing synaptic levels and impairing learning via saturated plasticity in adult rats (Uchimoto et al., 2014). SK2 channel levels are also ubiquitination-regulated. Deficiency of the E3 ubiquitin ligase UBE3A elevates postsynaptic SK2 by reducing its ubiquitination and endocytosis (Sun et al., 2020; Sun et al., 2015). Here, we observed significantly increased ubiquitinated/total SK2 ratio in the S1 region 24 h post-ketamine administration (Figure 5). Furthermore, consistent with prior reports that the proteasomal pathway is primarily responsible for SK2 degradation in rat CA1 (Müller et al., 2018), we found that the proteasome inhibitor MG132 elevated total SK2 levels in S1 of both control and ketamine-anesthetized rats and mitigated ketamine-induced SK2 reduction (Figure 6A). Furthermore, MG132 increased SK2 surface expression (Figure 6B), indicating proteasome inhibition promotes SK2 recycling to the membrane. These findings indicate that SK2 reduction results from increased ubiquitination and proteasomal degradation. Consequently, MG132 prevented the decrease in mAHP current amplitude in ketamine-treated rats (Figures 6C,D), as well as the associated changes in neuronal spike frequency (Figure 6E) and spike-frequency adaptation (Figure 6H). Apamin reversed these MG132 effects (Figures 6D,F,I), confirming elevated SK2 mediates MG132’s restoration of excitability and adaptation. Finally, MG132 inhibited the compensatory reduction in neuronal apoptosis (Figures 6J,K), providing additional evidence that the decrease in SK2 levels—and the consequent enhancement in pyramidal neuron activity—contributes to this compensatory apoptotic response.

While the use of MG132 strongly implicates the proteasome in the regulation of SK2 levels, we acknowledge certain limitations in interpreting these findings. As a broad-spectrum proteasome inhibitor, MG132 prevents the degradation of numerous cellular proteins and may induce general cellular stress, which could indirectly influence neuronal physiology. Furthermore, although previous work identifies the proteasome as a key degradation route for SK2 channels (Müller et al., 2018), as integral membrane proteins, endocytosed SK2 channels may also be subject to lysosomal degradation. Regarding the upstream mechanisms triggering this enhanced ubiquitination, the E3 ubiquitin ligase UBE3A represents a leading candidate. Recent evidence from Sun et al. (2020) suggests a sophisticated crosstalk between phosphorylation and ubiquitination in regulating SK2 trafficking. Specifically, PKA-mediated phosphorylation of the SK2 C-terminal domain is primarily involved in triggering its activity-dependent endocytosis, while UBE3A-mediated ubiquitination serves to predominantly inhibit the recycling of internalized SK2 channels back to the synaptic membrane. Crucially, PKA-mediated phosphorylation has been shown to facilitate UBE3A-dependent ubiquitination, suggesting that these two post-translational modifications collaboratively maintain optimal SK2 expression. We speculate that ketamine treatment may activate PKA to drive SK2 downregulation through an accelerated endocytosis-to-degradation sequence. Beyond reduced protein expression, impaired SK2 gating sensitivity may also drive post-ketamine hyperexcitability. SK channels are voltage-independent and gated solely by intracellular Ca^2+^ through a constitutively coupled calmodulin (CaM) complex (Allen et al., 2007). The Ca^2+^ sensitivity of this complex is fine-tuned by the opposing actions of channel-bound casein kinase 2 (CK2), which reduces affinity, and protein phosphatase 2A (PP2A), which restores it (Allen et al., 2007; Wang et al., 2018). Additionally, phosphatidylinositol bisphosphate (PIP2) can enhance SK2 activity by modulating CaM phosphorylation (Zhang et al., 2014). It is plausible that a functional downregulation of SK2 currents may occur alongside protein degradation, potentially synergizing to facilitate the increase in neuronal activity.

Collectively, this study identifies a previously unrecognized compensatory pathway within the developing brain that serves to balance anesthetic-induced and physiological apoptosis, and establishes SK2 channels as a critical molecular hub connecting neuronal activity to the apoptotic regulatory machinery. By identifying this internal defense mechanism, our findings offer a distinct perspective compared to traditional exogenous neuroprotective strategies. While exogenous interventions, such as neuroprotective peptides or pharmacological agents (e.g., lithium), aim to artificially block cell death (Yang et al., 2025; Zhang et al., 2025), our work highlights the brain’s inherent capacity for homeostatic resilience through post-translational ion channel modulation, specifically via the ubiquitin-proteasome system. These findings provide a robust preclinical framework for understanding the neurobiological underpinnings of anesthetic-induced neuroplasticity.

## Data Availability

The original contributions presented in the study are included in the article/supplementary material, further inquiries can be directed to the corresponding authors.
